# Foot health and quality of life in women with breast cancer undergoing chemotherapy: a cross-sectional study

**DOI:** 10.1186/s13047-023-00650-y

**Published:** 2023-08-21

**Authors:** Raquel Veiga-Seijo, Sonia Pertega-Diaz, Maria Eva Perez-Lopez, Lourdes Calvo Martinez, Silvia Antolin Novoa, Cristina Gonzalez-Martin

**Affiliations:** 1 Department of Health Sciences, Faculty of Nursing and Podiatry, Universidade da Coruña, Campus Esteiro, Ferrol, 15471 Spain; 2 Research Group in Nursing and Health Care, Instituto de Investigación Biomédica de A Coruña (INIBIC), Hospital Universitario de A Coruña (HUAC), Sergas, Universidade da Coruña (UDC), A Coruña, 15006 Spain; 3 Research Group in Rheumatology and Health (GIR-S), Faculty of Physiotherapy, Universidade da Coruña (UDC), Campus Oza, A Coruña, 15008 Spain; 4 Breast Unit, Medical Oncology Department, Hospital Universitario de A Coruña (HUAC), Instituto de Investigación Biomédica de A Coruña (INIBIC), Sergas, A Coruña, 15006 Spain

**Keywords:** Foot health, Podiatry, Quality of life, Breast cancer, Chemotherapy

## Abstract

**Background:**

Chemotherapy is one of the most widely used therapies for breast cancer, triggering important repercussions on people’s quality of life. However, little research has been undertaken about podiatric adverse effects. This study aimed was to determine the prevalence of podiatric pathology developed in people with breast cancer who receive chemotherapy.

**Methods:**

Observational, descriptive, and cross-sectional study was conducted in the Oncology service of the A Coruña University Hospital (northwest Spain). People with breast cancer and undergoing chemotherapy treatment of legal age (≥ 18), who signed the informed consent (*n* = 117) were included. Sociodemographic, comorbidity, disease and foot health variables, as well as two self-administered questionnaires (Foot Health Status Questionnaire and Foot Function Index) were studied. The current ethical-legal aspects were followed.

**Results:**

Foot health problems were highly prevalent, highlighting nail color changes (59.8%), onychocryptosis (39.7%), xerosis (62.4%), plantar fasciitis (12.8%), and neuropathic symptoms (75.2%). Some foot pain was presented in 77.8% of the sample, predominantly at nail level (15.4%) or sole of the foot and nail (14.5%). Most participants described their foot health as fair or poor (56.4%) and felt limited in walking (65.8%). The lowest score for the Foot Health Status Questionnaire was footwear (30.6(33.5)).

**Conclusions:**

Foot health adverse effects represent worrisome problems in women with breast cancer undergoing chemotherapy, due to their high prevalence and negative implications on quality of life. These problems are critical as they may have implications for stopping or reducing chemotherapy. All these results call for the development of more research to contribute to the care and wellbeing of people with cancer who receive treatments such as chemotherapy. Thus, this line of research is a new path to be developed by the podiatry community.

**Supplementary Information:**

The online version contains supplementary material available at 10.1186/s13047-023-00650-y.

## Introduction

 Cancer is a global threat to public health [1]. Different strategies and institutions attempt to provide preventive and health promotion measures to address this outstanding health challenge [2]. This is due to not only the important consequences for the quality of life and wellbeing of the person who suffers and their families [3]; but also, the significant impact on the health and socioeconomic systems [4, 5]. The magnitude of the problem must also be considered, since according to GLOBOCAN 2020 [6], there will be approximately 19.3 million new cases of cancer and 10.0 million deaths by the year 2040. Additionally, the COVID-19 represents a double challenge for the care process of people with cancer and triggers a further risk for their health [7, 8].

Worldwide, breast cancer is the most frequently diagnosed tumor [6]. In the case of women, it is the most common malignant tumor (47,8%), and it is estimated that 1 in 12 will develop it throughout their lives, making it the main cause of death [9, 10]. In Spain, there are an estimated 516,827 cases of this tumor in women in 2020. Regarding men, statistics agree that this tumour is infrequent [10–12].

Although there are progressively more therapeutic options for its treatment, chemotherapy continues to be one of the most used options to deal with this disease [12, 13]. The consequences that cancer and this treatment’s side effects have on people’s quality of life [3, 14] lead to the development of a person and family-centered approach [13]. This is due to the need to manage these effects and improve quality of life as well as survival rates, as a fundamental objective in the current Oncology field [15].

Chemotherapy’s side effects have been widely studied, including fatigue, nausea, vomiting, changes in appetite, nervous and muscular problems, weight changes, and emotional consequences [16]. In the case of breast cancer, taxane chemotherapy stands out because it plays a fundamental role in its treatment and produces side effects that affect body organs in which the foot is involved. Thus, this study particularly emphasizes foot health since previous research highlighted dermatological and neuropathic side effects [17], such as nail toxicities (40%) [18], hand-foot syndrome [19] or peripheral neuropathy (58.4%) [18, 20]. In fact, it has been highlighted the importance of extremities' effects on the quality of life in this population [18]. Besides, authors as Özdemir et al. [21] reported that women have a higher risk of experiencing adverse effects due to treatment, which is consistent with other studies [22, 23]. For instance, Barbu et al. [24], found out that female patients with dermatologic side effects had a significantly poorer quality of life than male patients, and Gusella et al. [25] observed that female have more adverse effects such as hand-foot disease than men. The above shows the relevance of establishing a starting point with this study population. Furthermore, the literature shows that little is known about how these therapies impact people’s foot health and, consequently, their quality of life, as a recent scoping review reported [26]. It is therefore essential to investigate these side effects in order to improve the quality of life of people with breast cancer undergoing chemotherapy.

Considering the gap in the literature on the exposed subject, this research aimed to determine the prevalence of podiatric pathology developed in people with breast cancer who receive chemotherapy. The specific objectives are: a) To estimate the prevalence of podiatric pathology (nail, skin, and biomechanical conditions), b) To explore peripheral neuropathy (symptoms of neuropathic origin and sensitivity alteration), and c) To determine foot and general health-related quality of life, as well as the functionality and pain of the foot.

## Methodology

### Study design and setting

An observational, descriptive, cross-sectional study was carried out prospectively in the Breast Unit and the Oncology Service of the A Coruña University Hospital of (northwest Spain), between May 2021 and January 2022.

### Sample size

The study included *n* = 117 participants. This sample size allowed us to determine the prevalence of podiatric pathology with an accuracy of ± 9% using a 95% confidence interval. As published prevalence estimates are not available, calculations were made using an estimated prevalence of 50% to maximise the sample size.

### Inclusion and exclusion criteria of the studied sample

The inclusion criteria of the sample were people diagnosed with early or metastatic breast cancer, of legal age (≥ 18), and who had received more than two cycles of chemotherapy or who had completed treatment in the last four months (adjuvant, neoadjuvant or palliative metastatic chemotherapy). Exclusion criteria were people with previous amputation of both lower limbs or who had not signed the informed consent after reading the participant information sheet and solving the doubts regarding the study.

### Data collection and variables

Once the ethical procedures were obtained, we proceeded with the consecutive recruitment of participants and the data collection, which was carried out by first author (qualified podiatrist and nurse) previously trained for this purpose. The study variables and measurements implied three well-differentiated phases:

- First. A general interview that included the study of sociodemographic variables (age, sex, gender, educational level, employment situation and family unit), toxic habits (smoking, alcohol consumption), anthropometric data (Body Mass Index (BMI), comorbidity (medication, general illnesses, Charlson Comorbidity Index [27]). Concerning breast cancer, variables related to cancer (date of diagnosis, type of tumor, main characteristics, general condition) and cancer treatment (drug and the number of cycles received) were studied. The morphological classification (ductal or lobular) and according to the molecular subtype (Luminal A, B HER 2 positive or negative, triple negative and HER2 positive) were recorded [28–30]. 
- Second. Podiatric examination in unloaded and standing position. The protocol was: a) visual assessment of nail, skin and structural conditions [31] following the Common Terminology Criteria for Adverse Events of the National Cancer Institute v.5 (NCI) [32, 33]; b) peripheral neuropathy exploration following the World Health Organization (WHO) scale [34]  and the Semmes–Weinstein Monofilament [35] to assess the sensitivity; c) the Foot Posture Index [36] and d) the study of the footprint, using the Clarke Angle, Staheli and Chippaux-Smirak Index, to categorize the footprint as cavus, normal or flat. The footprint was obtained by pedigraph [37] . Additional File 1 shows the definitions and diagnostic criteria for nail, structural and skin conditions, following what was described by Moreno de la Fuente et al. [31] , Miller et al. [32], and the National Cancer Institute [33].
- Third. Two validated and self-administered questionnaires: Foot Health Status Questionnaire (FHSQ) [38, 39] and the Foot Function Index (FFI) [40, 41]. The first is used to study the foot and general health-related quality of life. It presents 19 questions with a Likert-type scale. The first 13 questions are collected in 4 dimensions: foot pain (4 questions), foot function (4 questions), footwear (3 questions) and general foot health (2 questions). A score is obtained for each domain ranging from 0 (worst conditions) to 100 (best conditions). The second questionnaire contains 23 questions divided into 3 subscales: pain (9 items), disability (9 items) and functional or activity limitation (5 items). Each question is evaluated on a scale from 0 to 9 where 0 is no pain/difficulty, and 9 would mean the worst pain imaginable and/or such difficulty that help is required. High values indicate greater pain, disability, and activity limitation, and therefore a worse state of health.

### Ethical-legal aspects

This study was developed following the Declaration of Helsinki and current ethical-legal considerations. It received approval from the A Coruña-Ferrol Research Ethics Committee (2021/019). The entire sample received detailed information and explanation about the purpose and characteristics of this study. All participants provided written informed consent before being enrolled in the study.

### Statistical analysis

A descriptive analysis was performed, providing mean (standard deviation) for quantitative variables and frequencies and percentages for qualitative ones. Prevalence of podiatric pathology was determined together with 95% confidence intervals.

## Results

### Sociodemographic and clinical characteristics

One hundred seventeen participants have been recruited and studied. The general and disease characteristics are shown in Table 1. Mean age was 53.3(12.1) years. All the studied sample were women and reported belonging to the female gender. Regarding the BMI, 41.0% had a normal weight, 29.9% were overweight, and 25.6% obesity. Regarding the disease and previous comorbidity, 36.8% had cardiovascular diseases, 23.1% neurological, and 11.1% oncological. In respect of breast cancer familiar history, 47.4% had it, with a second-degree relationship (23.1%).

**Table 1:** General and clinical characteristics of the sample studied (*n* = 117)

	**Mean (SD)**	**Median (Min–Max)**
**Age (years)**	53,3 (12,1)	50,0 (30–88)
**BMI ( (kg/m**^**2**^**)**	26,4 (5,0)	25,5 (17,3–38,2)
**Charlson Comorbidity Index**	4,4 (2,1)	6,0 (0–8)
**Age-Adjusted Charlson Comorbidity Index**	5,1 (2,5)	6 (0–11)
**Time of evolution of cancer (months)**	11,2 (18,5)	(2–139)
	**n (%)**	**95% CI**
**Civil status**		
Single	22 (19,0)	11,3–26,3
Married / Co-habitant	78 (67,2)	57,7–75,6
Widow	8 (6,9)	1,8–11,8
Divorced / Separated	8 (6,9)	1,8–11,8
**Education level**		
No studies	4 (3,4)	0,9–8,5
Primary studies	15 (12,9)	6,3–19,3
Secondary/Higher Education	60 (51,7)	41,8–60,8
University studies	37 (31,9)	22,7–40,5
**Employment situation**		
Active	4 (3,4)	0,9–8,5
Unemployed	5 (4,3)	1,4–9,7
Pension	16 (13,8)	7,0–20,3
Sick leave	67 (57,8)	47,9–66,6
Inability to work	5 (4,3)	1,4–9,7
Household	14 (12,1)	5,6–18,3
**Smoking habit**		
Yes	8 (6,9)	1,8–11,8
Former smoker	33 (28,4)	19,6–36,8
**Breast cancer**		
Invasive ductal	107 (92,2)	85,9–96,9
Invasive lobuar	9 (7,8)	2,4–12,9
**Breast Cancer Subtype**		
Luminal A	2 (1,7)	0,2–6,0
Luminal B HER2 negative	54 (46,2)	36,7–55,6
Luminal B HER2 positive	33 (28,2)	19,6–36,8
Triple Negative	21 (17,9)	10,6–25,3
HER 2 positive	7 (6,0)	1,2–10,7
**Metastasis**	59 (50,4)	39,2–58,2
**Cancer therapy**		
Chemotherapy or after finishing it	82 (70,2)	61,4–78,8
Anti-HER2 Therapy after chemotherapy	11 (9,4)	3,7–15,1
Chemotherapy + Anti-HER 2 Therapy	11 (9,4)	3,7–15,1
**Number of cycles received at time of visit**		
4 cycles	34 (29,1)	20,4–37,7
5 cycles	17 (14,5)	7,7–21,3
6 cycles	11 (9,4)	3,7–15,1
8 cycles	25 (21,4)	13,5–29,2
Others	30 (25,6)	17,3–34,0

*SD* Standard Deviation, *CI* Confidence Interval, *Min* Minimum; *Mx* Maximum, *BMI* Body Mass Index

**Fig. 1:**
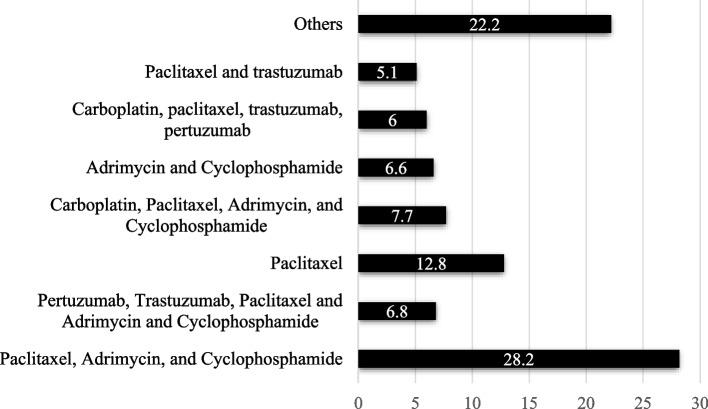
Most common chemotherapy treatment (%)

Most of the women were in stage IIA (27.4%) or IIB (22.2%), with 16.2% in stage IV. The most frequent Nottingham Scale classification was grade 3 (51.3%) and 2 (45.3%). Regarding the ECOG scale, 77.8% presented a score 1. Almost all women (97.4%) reported asthenia (68.4% grade 2; 28.9% grade 3). The treatment plan was mostly neoadjuvant (62.4%). Figure 1 shows the most common chemotherapy treatments that the sample studied had received at the time of the visit. The mean number of cycles received at the time of the visit was 6.8(4.1). In addition, 91.5% received surgery for their disease process and 61.5% radiotherapy.

### Foot health problems: structural, nail, skin, and neuropathic origin

Concerning the structural pathology of the forefoot, 85.5% of the sample studied had one of them. The hallux valgus was the most prevalent pathology (74.4%), followed by claw toes (53.0%) (Table 2). Regarding the hindfoot, 12.8% developed plantar fasciitis. Besides, most of the participants studied had at least one nail pathology (91.5%) and/or skin pathology (88.0%). Presenting some color change in the nail plate was the most prevalent nail pathologies (59,8%), such as diffuse melalonychia (41.9%). With regard to skin pathology, it is worth highlighting problems associated with dryness and higher-pressure points, such as xerosis (62.4%) and hyperkeratosis (65.8%). In addition, the hand-foot syndrome was present in 35.9% of the people studied.

**Table 2:** Structural, nail and skin foot pathology (*n* = 117)

	**n (%)**	**95% CI**
**Structural Pathology Forefoot and Hindfoot**	**100 (85,5)**	78,6–92,3
Edema	43 (36,8)	27,6–45,9
Morton's Neuroma	3 (2,6)	0,5–7,3
Claw toes	62 (53,4)	43,5–62,5
Hallux Extensus	6 (5,1)	0,7–9,5
Hallux Limitus	27 (23,1)	15,0–31,3
Second Finger Supraduct	14 (12,0)	5,6–18,3
Hallux Valgus (Manchester Scale)	87 (74,4)	66,0–82,7
Grade 1: no deformity	30 (25,0)	17,3–34,0
Grade 2: mild deformity	47 (40,5)	30,9–49,5
Grade 3: moderate deformity	39 (33,6)	24,4–42,3
Grade 4: severe deformity	4 (3,4)	0,9–8,5
Plantar Fasciitis	15 (12,8)	6,3–19,3
Calcaneal Spur	2 (1,7)	0,2–6,0
Achilles Tendinitis	4 (3,4)	0,9–8,5
**Nail Pathology**	**106 (91,5)**	84,9–96,3
Subungual Hematoma	7 (6,0)	1,3–10,7
Leukonychia	7 (6,0)	1,3–10,7
Onychoatrophy	6 (5,2)	0,7–9,5
Onychodystrophy	8 (6,9)	1,8–11,8
Onychogryphosis	22 (19,0)	11,3–26,3
Onychocryptosis	46 (39,7)	30,0–48,6
Onychocolosis	44 (37,9)	28,4–46,8
Onychomadesis	13 (11,2)	5,0–17,2
Nail Fall	6 (5,2)	0,7–9,5
Paronychia	15 (12,9)	6,3–19,3
Beau Lines	18 (15,5)	8,4–22,3
Subungual Hyperkeratosis	31 (26,5)	18,1–34,9
Terry Nails	7 (6,0)	1,3–10,7
Half and Half Nails	22 (19)	11,3–26,3
Color Change	70 (59,8)	50,5–69,1
Diffuse Melalonychia	49 (41,9)	31,7–50,4
Longitudinal Melalonychia	14 (12,0)	5,0–17,2
Onychorrhexis	8 (6,8)	1,3–10,7
Splinter Hemorrhage	9 (7,7)	2,4–12,9
Anonychia	7 (6,0)	1,3–10,7
Yellow Nail Syndrome	16 (13,7)	7,0–20,3
**Skin Pathology**	**102 (87,9)**	80,7–93,7
Xerosis	73 (62,4)	52,3–70,8
Hyperkeratosis	77 (65,8)	56,8–74,8
Heloma	17 (14,5)	7,7–21,3
Cracks	17 (14,5)	7,7–21,3
Hand-Foot Syndrome	41 (35,9)	26,0–44,1
Ulcers or wounds	6 (5,2)	0,7–9,5
Blisters	17 (14,5)	7,7–21,3
Erythema	26 (22,2)	12,8–28,2
Fragile skin	27 (23,1)	15,0–31,1
Skin atrophy	10 (8,5)	3,0–14,0
Peeling	17 (14,5)	7,7–21,3

*CI* Confidence Interval

With respect to the study of peripheral neuropathy and associated symptoms (Table 3), it is noteworthy that 56.0% reported having tingling in the feet, 25.6% stiffness and 7.7% a feeling of imbalance because of chemotherapy treatment. According to the WHO Scale for the study of peripheral neuropathy, 38.5% presented a grade 2, which implies that the pain interfered with the person’s functionality. Besides, the assessment of sensitivity using monofilament showed that about 25% had no sensitivity in any of the points studied.

**Table 3:** Peripheral neuropathy and sensitivity (*n* = 117)

	**n (%)**	**95% CI**
**Symptoms**	**88 (75,2)**	**67,0–83,5**
Tingling/Crawling sensation	65 (56,0)	46,1–65,0
Abrasion	8 (6,8)	1,8–11,8
Needles	23 (19,7)	12,0–27,3
Imbalance	9 (7,7)	2,4–12,9
Numbness, lack of sensitivity	17 (14,5)	7,7–21,3
Muscular weakness	18 (15,4)	8,4–22,3
Pins	15 (12,8)	6,3–19,3
Muscle and joint stiffness	30 (25,6)	16,5–33,0
Dysesthesia-Paraesthesia	20 (17,1)	9,8–24,3
**WHO Peripheral Neuropathy Scale**		
No symptoms	29 (24,8)	18,8–35,8
Paraesthesia or weakness without pain or loss of function	25 (21,4)	9,1–23,3
Pain that interferes with function, but not with ADLs	45 (38,8)	29,2–47,7
Pain that interferes with ADLs	17 (14,5)	7,7–21,3
Motor affectation and/or disabling pain	1 (0,9)	0,02–4,7
**HFS Study Degree**		
**WHO HFS Scale**		
Grade 1	6 (14,3)	2,5–26,1
Grade 2	10 (23,8)	9,7–37,9
Grade 3	14 (33,3)	17,9–48,8
Grade 4	12 (28,6)	13,7–43,4
**NCI HFS Scale**		
Grade 1	6 (14,3)	2,5–26,1
Grade 2	16 (38,1)	22,2–54,0
Grade 3	20 (47,6)	31,3–63,9
**Sensitivity assessment using monofilament**		
First Toe	30 (25,9)	17,3–34,0
First Metatarsal Head	29 (25,0)	16,5–33,0
Third Metatarsal Head	29 (25,0)	16,5–33,0
Fifth Metatarsal Head	26 (22,4)	14,3–30,2

*CI* Confidence Interval, *WHO* World Health Organization, *NCI* National Cancer Institute, *HFS* Hand Foot Syndrome, *ADLs* Activities of Daily Living

The Foot Posture Index (Table 4) reveals that the majority had a pronated foot position (67.5% left foot and 66.7% right foot). Regarding the footprints, according to the Clarke and Chippaux-Smirak angle, they were mostly normal (62.9% left, 62.1% right, and 62.1% left and 71.6% right, respectively). Considering the Staheli Index, the majority were cavus (56.4% left and 49.1% right).

**Table 4:** Foot Posture Index and Plantar Footprint Study

	**Left foot**		**Right foot**	
	**n (%)**	**95% CI**	**n (%)**	**95% CI**
**Foot Posture Index (** ***n*** ** = 117)**				
Supination	4 (3,4)	0,9–8,5	4 (3,4)	0,9–8,5
Neutral	34 (29,1)	20,4–37,7	35 (29,9)	21,2–38,6
Pronation	79 (67,5)	58,6–76,4	78 (66,7)	57,7–75,6
**Footprint Study (** ***n*** ** = 116)**				
**Clarke Angle**				
Cavus (< 31º)	27 (23,1)	15,1–31,4	37 (31,9)	23,0–40,8
Normal (31º a 45º)	73 (62,9)	53,7–72,1	72 (62,1)	52,8–71,3
Flat (> 45º)	16 (13,8)	7,1–20,5	7 (6,0)	7,1–20,5
**Chippaux-Smirak Index**				
Cavus (> 25%)	29 (25,0)	16,7–33,3	21 (18,1)	10,7–25,5
Normal (25% – 45%)	72 (62,1)	52,8–71,3	83 (71,6)	62,9–80,2
Flat (> 45%)	15 (12,9)	6,4–19,5	12 (10,3)	4,4–16,3
**Staheli Index**				
Cavus (< 0,6)	66 (56,4)	47,4–66,3	57 (49,1)	39,6–58,7
Normal (0,6 – 0,69)	24 (20,7)	12,9–28,5	28 (24,1)	15,9–32,3
Flat (> 0,69)	26 (22,4)	14,4–30,4	31 (26,7)	18,2–35,2
**Foot Hyperpressure (** ***n*** ** = 116)**				
1st toe	32 (27,6)	19,0–36,1	38 (32,8)	23,8–41,7
1st metatarsal head	56 (48,3)	38,7–57,8	61 (62,6)	43,1–62,1
2nd metatarsal head	61 (52,6)	43,1–62,1	65 (56,0)	46,6–65,5
3rd metatarsal head	61 (52,6)	43,1–62,1	62 (53,4)	43,9–63,0
4th metatarsal head	49 (42,2)	32,8–51,7	50 (43,1)	33,7–52,5
5th metatarsal head	46 (39,7)	30,3–49,0	46 (39,7)	30,3–49,0
Heel	19 (16,4)	9,2–23,5	15 (12,9)	6,4–19,5
Internal Part Heel	28 (24,1)	15,9–32,3	34 (29,3)	20,6–38,0
External Heel	11 (9,5)	3,7–15,2	10 (8,6)	3,1–14,2

*CI* Confidence Interval

### Foot health, quality of life, pain, and functionality

Foot health, health-related quality of life, foot functionality and pain were studied with the FHSQ and FFI questionnaires (Table 5). Regarding pain, 77.8% presented some degree of pain in their feet. On most occasions, the pain was at nail level (15.4%), at plantar and nail level (14.5%), in the forefoot (12.8%), at plantar level (11.1%) and metatarsal (9.4%).

**Table 5:** Foot health, functionality, foot pain and health-related quality of life

	**Mean (SD)**	**Median (Min–Max)**
**FFI**	30,4 (19,6)	28,0 (8,5–93,2)
**Items of FFI**		
Foot pain at the worst time	5,0 (2,7)	5,0 (1–10)
foot pain at the end of the day	4,8 ( 2,7)	5,0 (1–10)
**FHSQ**		
Foot pain	64,0 (23,9)	68,7 (0–100,0)
Foot function	76,0 (22,8)	75,0 (6,25 – 100)
Footwear	30,6 (33,5)	16,7 (0,0 – 308,3)
General health	34,6 (16,7)	25,0 (0,0 – 75,0)
	**n (%)**	**95% CI**
**Items of FHSQ**		
**Degree of foot pain in the past week**		
Very mild or none	47 (40,2)	30,9–49,5
Mild	29 (24,8)	16,5–33,0
Moderate or severe	41 (35,0)	26,0–44,1
**Frequency of sharp pain**		
Ocasionally or Never	66 (56,4)	47,0–65,8
Fairly Many Times	17 (14,5)	7,7–21,3
Very often or Always	34 (32,1)	20,4–37,7
**Limitation to walk due to foot health**		
Slightly or Not at All	84 (71,8)	63,2–80,4
Moderately	15 (12,8)	6,3–19,3
Quite a bit or Extremely	18 (15,4)	8,4–22,3
**General foot health status rating**		
Excellent or Very Good	3 (2,6)	0,5–7,3
Good	48 (41,0)	31,7–50,4
Fair or Poor	66 (56,4)	47,0–65,8
**Difficulty finding shoes that do not hurt**		
Strongly Agree or Agree	68 (58,1)	48,7–67,5
Neither Agree nor Disagree	7 (6,0)	1,3–10,7
Disagree or Strongly Disagree	42 (35,9)	26,8–45,0
**Limitation of health status in performing vigorous activities**		
Not Limited at All	11 (9,4)	3,7–15,1
Limited a Little	61 (52,1)	42,7–61,6
Limited a Lot	45 (38,5)	29,2–47,7
**Limitation of health status in moderate activities, such as cleaning, walking…**		
Not Limited at All	13 (11,1)	5,0–17,2
Limited a Little	72 (61,5)	52,3–70,8
Limited a Lot	32 (27,4)	18,8–35,8
**Interference of your health or emotional problems with your social activities**		
Slightly or Not at All	82 (70,1)	61,3–78,8
Moderately	21 (17,9)	10,6–25,3
Quite a bit or Extremely	14 (12,0)	5,7–18,3

*SD* Standard Deviation, *FFI* Foot Function Index, *FHSQ* Foot Health Status Questionnaire, *CI* Confidence Interval

According to the FHSQ domains, the domain with the highest average score was foot function (76.0(22.8)), and the domain with the lowest score was footwear (30.6(33.5)). Likewise, 20.5% received pharmacological treatment for neuropathic pain, 17.1% topical foot creams, and 6.8% required oral antibiotics due to foot infections. In addition, it is important to note that 45 people needed to delay or stop chemotherapy treatment and 2 to reduce their dose. Of this number of cases, 17 people (more than a third) had these changes in their treatment due to foot health problems.

Another highlighted point was that 50.4% presented difficulties in finding shoes that do not trigger pain. Likewise, 56.4% participants identified their foot health as fair or poor, and felt limited in walking (65.8%).

Finally, the majority had limitations to carry out intense efforts (90.6%) and activities such as cleaning, walking and day-to-day activities (88.9%). In addition, 60.7% of the sample felt tired and/or exhausted (59.8%) almost always.

## Discussion

This study’s purpose was to determine the foot health, functionality, pain, and health-related quality of life, in women with breast cancer undergoing chemotherapy treatment.

The state of the art showed inconclusive and little knowledge about the podiatry pathology of cancer patients undergoing chemotherapy. Therefore, to the best of our knowledge, this is the first comprehensive and holistic research aimed at studying the adverse effects that occur in foot health, emphasizing participants’ perceptions about their foot health, quality of life and functional capacity. Findings show the relevance of this issue and the need to consider foot health in the field of Oncology. Even though nail and dermatological foot problems happen in the overall population, this condition is commonly seen in this research. While it is true that not all the pathologies studied are due to chemotherapy treatment (such as hallux valgus), it is known that certain nail and skin pathologies, as well as peripheral neuropathy, are due to cancer therapies. Therefore, and in addition to the fact that they may have previous podiatric problems, the relevance of an interdisciplinary care team in the field of Oncology that also includes Podiatry is justified. This study demonstrates an innovative and promising line of research that must be considered by the podiatry community in order to develop an emergent care framework for people with cancer, working towards a new research and clinical agenda that contributes to a holistic care approach from a podiatric perspective.

A better understanding of the importance of this topic starts from being aware that adequate foot health allows for walking and leading an active lifestyle [42], and is therefore a determinant of health [43]. To date, the scientific evidence focused on the changes that the feet can undergo in relation to self-care habits, work circumstances or chronic diseases such as rheumatoid arthritis [44, 45], diabetes [46] and psoriasis [47], leaving the field of Oncology blurred. On top of this is the fact that 2 out of 3 people with breast cancer still receive chemotherapy nowadays [12], unleashing important consequences on the quality of life of the people who receive it. Thus, managing its side effects and improving quality of life at the same time that survival rates are the main objective of the Oncology field today.

### Structural, skin, nail conditions and peripheral neuropathy

The present research adds new information to the epidemiology knowledge. Findings show that nail and skin problems have been very prevalent side effects in this research. However, there are not enough investigations that inform concretely about these foot problems and their implications on the quality of life and wellbeing from the discipline of Podiatry or others. Only one review and clinical experience [48] was found, which showed the main foot adverse effects, providing the most interesting characteristics of the most relevant ones from the clinical point of view.

Likewise, some adverse effects have been studied, as peripheral neuropathy, hand-foot syndrome or nail toxicities, although they were addressed independently and, sometimes, without specifying its manifestation in the foot. This is the case of Biswal and Mehta’s study [49], who concluded that 78.6% of the sample presented some skin problem in some part of the body. The foot is only identified when the hand-foot syndrome is mentioned, present in 2.6%. Xerosis was only present in 4.4% and melalonychia in 2.9%. These data differ from our findings. Another study [50] indicates a prevalence of 35.3% for the hand-foot syndrome, consistent with the present investigation. They also found 14.1% of xerosis, and 12% of nail changes, which do not match with our findings. On the other hand, the research by Hackbarth et al. [51] identified a prevalence of 18.7% for hand-foot syndrome and 23.1% for nail changes, but it is not specified whether or not they occur in the foot. The same situation occurs in Zawar et al.’s study [52] who only focus on nail problems, but without specifying how they manifest in the feet. Another investigation [53] also focused solely on nail changes associated with docetaxel, where they observed that the 45.5% involved the foot, which implied problems finding shoes in 37% of cases. This result differs from what was obtained in the present investigation, with a prevalence of 91.5%.

In addition to the above, it is essential to identify and manage skin problems, not only so that the quality of life of people with cancer is ensured [18], but also so that modifications in treatment doses are minimal, as indicated by scientific evidence [17]. The present study reveals that of the people who had their treatment dose suspended or reduced, 38.6% was due to problems with their feet, which indicates that this aspect is not yet assured. In this sense, Azuma et al. [54] call for the need to adjust the dose early based on the stage of the hand-foot syndrome, as it can improve the efficacy of the treatment. Also, Gresset, Stanford and Hardwicke [55] show the relevance of not only reducing or stopping treatment, but also developing support measures to reduce pain and discomfort, as well as prevent secondary infections. On the other hand, Biswal and Mehta [49] refer to the fact that the diagnosis of skin problems in this group is a challenge, due to the complexity of the disease process and the existence of combined protocols used for treatment.

In relation to peripheral neuropathy, this side effect has been widely described in people with cancer. There are several factors associated with chronic painful neuropathy: being born premature, having a lower income, a higher number of comorbidities, and/or back pain. Age, diabetes, alcohol, BMI and type of chemotherapy also play a role. Gender, education, marital and smoking have not been associated [56]. Despite this, there is no research that attempts to find out how this problem triggers greater biomechanical or other problems in people who suffer from it. Vizcaíno et al. [57] studied neuropathy at a global level and its implication for quality of life. They reveal that most of the pain was located in the hands and feet, and that it impacted all daily life activities. Likewise, they have considered this problem as the most important compared to other adverse ones, from the participants’ subjective perspective. Besides, grade 2 and 3 have been the most predominant (36% and 29%, respectively), which agrees with our findings. Regarding sensitivity, Zhi et al. [58] indicated that people with moderate to severe neuropathy have implications for tactile and vibratory sensitivity. In this research, tactile sensitivity has been affected in about 25% of the sample studied, while, in their study, it has not reached 4%.

Regarding the discussion of this research on structural and/or biomechanical pathologies, only one study [59] refers to gait and balance in people with chemotherapy-induced peripheral neuropathy, where they found problems with walking speed and balance, affecting the person's daily routines.

### Foot health repercussions on the quality of life and daily activities

The negative repercussions of foot health problems on quality of life have been widely studied in the literature in other populations. For instance, Lopez-Lopez et al. [60] pointed out that foot problems have a negative impact on the general population’s quality of life. In the case of people with breast cancer, only one research article indicates that they have low foot health related to the quality of life [61]. The domains of foot pain, foot function, footwear, and general health show similar results to this study. This emphasizes the need to pay more attention to this problem.

The deterioration of foot health and its corresponding quality of life is also associated with aging [62]. This should be highlighted in this study since the population studied has a young average age, in which no podiatric problems are expected, so a decrease is added to their wellbeing. On the other hand, in relation to sex, it is known that in the general population, women have a higher risk of experiencing foot health problems [63]. This study focused on breast cancer as it represents a problem with a high socio-economic magnitude [10–12], the chemotherapy treatments commonly employed have adverse effects that affect the foot [16, 17, 19, 20], and in addition, women are at a higher risk of experiences reactions due to cancer therapies [21]. However, the critical review of the literature and the present field study suggests the need for further research including both sexes and adopting a gender perspective to the study of the effects and their implications on QoL.

In addition, other studies have already indicated the associations of foot health conditions with psychological and emotional problems, increasing scores of stress and depression, being a highly relevant field of study [64]. Likewise, it is important to consider the significant effect that physical activity has on the emotional and psychological health of people with breast cancer. Therefore, it can be assumed that without proper foot health, this may be unattainable [65]. This is consistent with the present investigation, where a high and diverse prevalence of foot conditions were found, as most people found themselves limited to walk, or mostly presented a self-perceived not so good or poor foot health.

Finally, different toxicities that have been well studied in the literature present relevant implications on quality of life, and most of them have repercussions on foot health. This is the case of the hand-foot syndrome, described as the adverse effect that most strongly impacts the quality of life among the cutaneous effects described [66]. This result is consistent with another study, which indicates that foot symptoms restrict daily activities by 44.7% [19]. Another condition is chemotherapy-induced peripheral neuropathy, which is also reported to be associated with impaired quality of life, functionality, and personal finances, according to a recent study [67].

### Study limitations

This study has several limitations that must be considered. Firstly, the sample size may be limited, although it has been calculated to determine the prevalence of foot problems in the study population. It is important to consider that the data collection took place during the COVID-19 pandemic, and that the population studied is characterized by special vulnerability due to the effects of treatment on their health. In addition, no man was part of the study sample, although this was not an exclusion criteria, and further studies with both sexes need to be developed. In addition, given that the included participants received anthracyclines, taxanes and capecitabine, further evaluation should address other drugs. On the other hand, this is a cross-sectional study, in which the podiatric pathologies that the people presented at the beginning of the study are not indicated. Mixed methodologies and longitudinal studies are necessary to create further understanding of the topic addressed. Although most of the found problems are identified as adverse effects as a consequence of cancer therapies, novel data provided by this study on structural pathologies and biomechanics of the foot cannot be fully defined due to the treatment received, and further studies may be required.

### Future research perspectives

The present investigation calls us to develop and prioritize the podiatric adverse effects in people undergoing cancer therapies and their implications on the quality of life. In this sense, it has not yet been possible to compare the prevalence of podiatric pathology since no study with the same objective has been found. Therefore, it is necessary to develop more research, and to include other tumor types, both sexes, the gender perspective, other geographical locations, and ethnicities. In addition, advancing in this knowledge will help health professionals to pay more attention and reflect on the consequences and significant impact that it can have on general health, such as the possibility of walking, moving, and maintaining an active and healthy life. Accordingly, this research calls to reflect in a new concept on the foot health adverse effects due to cancer therapies. Also, future studies should also be directed towards developing foot health assessment scales, that include the impact of adverse effects on biomechanics and gait, since the findings of this study suggested.

As previously indicated, future research should adopt mixed methodologies and longitudinal studies, in order to advance research in this field. The present research is the first phase of a larger project. These findings indicated the relevance of conducting a longitudinal and qualitative methodology, which is expected to be published in the future. Future research should also focus on the chemotherapy’s long-term effects on foot health, considering the survival rate of people with breast cancer.

Finally, the gap in the scientific evidence is linked to the lack of guidelines in clinical practice and the need for attention that people with cancer have regarding their foot health. Therefore, it is necessary to move forward in this field and to be able to contribute to the quality of life, which can be enhanced through foot health care. For this purpose, it is necessary to pay attention to foot health in the Oncology service. On this point, it is important to remember that integral attention of the person must be ensured, since the support to the adaptation to the disease is a fundamental aspect of the physical, social, and emotional wellbeing, as indicated by different authors and strategies such as Sustainable Development Goals [14]. The podiatry community needs to start getting involved in these agendas.

## Conclusions

Podiatric problems represent an important health problem in women with breast cancer who receive chemotherapy treatment. The high prevalence of nail, skin, biomechanical and neuropathic disorders is striking, being the cause in more than a third of the sample of the reduction or suspension of treatment.

The results presented call for further research to contribute to the care and wellbeing of people with cancer undergoing treatments such as chemotherapy. Thus, this exploratory research starts a new path to continue improving podiatry knowledge in this field.

### Supplementary Information

**Additional file 1. **Definitions and diagnostic criteria for structural, nail and skin conditions. 

## Data Availability

Request for further details of the data set and queries relating to data sharing arrangements may be submitted to Raquel Veiga-Seijo (raquel.veiga.seijo@udc.es). The data that support the findings of this study are not openly available due to them containing information that could compromise research participant privacy/consent.

## Abbreviations

BMI: Body Mass Index
FFI: Foot Function Index
FHSQ: Foot Health Status Questionnaire
NCI: National Cancer Institute
WHO: World Health Organization
