# Supplementary material for: Foot health and quality of life in women with breast cancer undergoing chemotherapy: a cross-sectional study
Source: J Foot Ankle Res. 2023 Aug 21;16:52. doi: 10.1186/s13047-023-00650-y (PMC10440865; doi:10.1186/s13047-023-00650-y)
Supplement: Supplementary file 1 — Additional file 1. Definitions and diagnostic criteria for structural, nail and skin conditions. [file 13047_2023_650_MOESM1_ESM.docx]

**Additional File 1. Definitions and diagnostic criteria for structural, nail and skin conditions**

| Term | Definition |
| --- | --- |
| Structural pathology | |
| Morton’s Neuroma | Compressive neuropathy that mainly affects the digital nerves of the lower limbs. Only those cases in which its presence has been suspected through the symptoms of the person and the examination with Mulder's sign are included, as no complementary test has been carried out to confirm this pathology. |
| Claw toes | Deformity in the sagittal plane, in which the metatarsophalangeal joint is in extension, and the proximal and distal interphalangeal joints are in flexion. |
| Hallux Extensus | Permanent extension of the two phalanges of the first toe. |
| Hallux Limitus | Limitation of dorsal flexion of the first metatarsophalangeal joint. |
| Second Finger Supraduct | Lateral deviation of the second toe above the first toe. |
| Hallux Valgus | It was assessed considering the Manchester Scale. It is a static subluxation of the first metatarsophalangeal joint, with medial deviation of the first metatarsal and lateral deviation (in abduction and valgus) of the hallux with respect to the midline of the body. It consists of a primarily osteoarticular lesion with associated soft tissue lesions and additional deformities of the following toes. |
| Plantar fasciitis | Inflammation and localised degeneration of the plantar aponeurosis. It is mainly characterised by pain, which is usually located on the medial aspect of the calcaneus and is often accompanied by inflammation. |
| Calcaneal Spur | Triangular or spear-shaped bony growth of the calcaneal bone. |
| Achilles Tendinitis | Tendinitis of the Achilles tendon. |
| Nail pathology | |
| Subungual Hematoma | Accumulation of blood under the nail plate. It can cause very accentuated pain due to the pressure produced under the nail. If the trauma is recent, a blue-violet colouring is observed. After two or three days of evolution, a brownish-black colour caused by blood coagulation will appear. |
| Leukonychia | A white pigmentation of the nail that can be seen partially or completely, congenital, or acquired, and real or apparent. |
| Onychoatrophy | Decrease in the development of the nail in size, thickness, and texture. It is thinned, brittle and hollowed out in the centre hollowed out in the centre (spoon-shaped). |
| Onychodystrophy | This is an alteration in the morphology of the nail regardless of its origin. |
| Onychogryphosis | This is a hypertrophy of the nail that causes detachment from the nail bed and over-elevation, adopting a horn shape with transverse grooves on its surface. |
| Onychocryptosis | The edge of the nail becomes embedded in the soft tissues, which in advanced cases causes an inflammatory reaction with added superinfection. |
| Onychocolosis | This is a detachment of the nail from the nail bed. It begins at the distal edge of the nail, an area that takes on a whitish colour. |
| Onychomadesis | This is a detachment of the nail. |
| Paronychia | Bacterial infection of the soft parts located on both sides and base of the nail. The nail plate is not involved in this process. |
| Beau Lines | These are deep transverse grooves affecting the nail matrix. It is due to mitotic disruption of the proximal matrix. |
| Subungual Hyperkeratosis | Accumulation of hyperkeratosis under the nail plate, usually in the hyponychium. |
| Terry Nails | Leukonychia affects the entire nail plate and leaves a distal band of 1-2 mm free. |
| Half and Half Nails | Leukonychia affects the proximal half of the nail. |
| Melanonychia | It is characterised by a dark colour due to an increase in the number of melanocytes. It may be diffuse or take on a longitudinal appearance. Chromonychia induced by antineoplastic drugs has different forms, of which melanonychia is the most common form, and may be diffuse, transverse or longitudinal. |
| Onychorrhexis | Nails with longitudinal grooves. |
| Splinter Hemorrhage | These are dark red longitudinal subungual. Their shape is due to the longitudinal orientation of the capillaries in the nail bed. |
| Anonychia | Absence of the nail. |
| Yellow Nail Syndrome | Yellow discolouration with increased transverse curvature, thickening and opacity of the lamina, slowing or arrest of nail growth, as well as loss of cuticles. |
| Skin pathology | |
| Xerosis | Abnormal dryness of the skin and other mucous membranes. |
| Hyperkeratosis | Hypertrophy or hyperplasia of the corneal layer, showing thickening due to a considerable increase in epidermal cells, especially keratinocytes, which show an increase in keratin. |
| Heloma | Keratopathy with a core that corresponds to intermittent pressure of the skin on a bony point, which causes ischaemia in the basal layer of the epidermis. |
| Cracks | A cutaneous fissure that forms in the skin of various parts of the body or in mucous membranes next to the skin. |
| Hand-Foot Syndrome | Side effect of some cancer treatments, resulting in redness, swelling and pain in the palms of the hands and feet, with occasional blistering and difficulty walking. |
| Blisters | Local elevation of the epidermis due to accumulation of fluid, caused by rubbing, heat, or skin diseases. Also called cutaneous vesicle. |
| Erythema | Reddening of the skin. |
| Fragile skin | The structure and function of the epidermal barrier are affected, leading to skin barrier dysfunction. It usually has a lower constitutional threshold of resistance to environmental stresses. |
| Skin atrophy | The structures that make up the skin diminish in middle age. Their thickness and proper functionality decreases, especially in relation to the elastic fibres. |
| Peeling | Loss of the outermost layer of the skin. |
